# Chronic tophaceous gout

**DOI:** 10.11604/pamj.2018.30.64.15292

**Published:** 2018-05-28

**Authors:** Petros Ioannou, Symeon Panagiotakis

**Affiliations:** 1 Internal Medicine Department, University Hospital of Heraklion

**Keywords:** Uric acid, tophi, arthritis, geriatrics, colchicine

## Image in medicine

A 98-year old caucasian woman with a history of gout presented to the emergency department due to diarrhea and joint pain. Her chronic medications included allopurinol and colchicine. Physical examination revealed abdominal tenderness without rebound tenderness and normal bowel sounds, as well as enlarged, deformed, painful digits at the upper and lower limbs. The laboratory exams revealed leukocytosis, an elevated Erythrocyte Sedimentation Rate of 82mm at the 1^st^ hour and a serum uric acid of 8.4mg/dl. The patient was admitted due to presumed colchicine toxicity and uric acid arthritis and was treated with intravenous hydration and corticosteroids with improvement of the arthritis and the diarrheas. Uric acid is produced from the purine metabolism after the breakdown of the nucleic acids that are produced after cell destruction and exogenous intake. Since uric acid is the end product of this metabolic pathway, its concentration in the plasma reflects a balance between production and renal excretion; however, about 10% of people are genetically predisposed to have increased uric acid concentration and are predisposed to uric acid nephropathy, nephrolithiasis, gout and development of tophi, like in our patient. Tophi are deposits of monosodium urate in the synovial or soft tissues and in the bones near the joints. Their presence signifies the presence of gout, the commonest inflammatory arthritis in elderly in the Western world. Rarely, tophi may lead to skin ulceration and discharge of chalky uric acid crystals through the skin.

**Figure 1:**
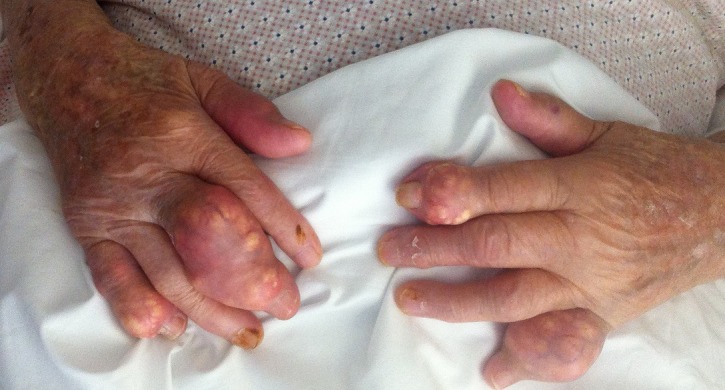
Shown are the enlarged, deformed and painful digits at the upper patient’s limbs

